# Identification and characterization of the peroxin 1 gene *MoPEX1* required for infection-related morphogenesis and pathogenicity in *Magnaporthe oryzae*

**DOI:** 10.1038/srep36292

**Published:** 2016-11-08

**Authors:** Shuzhen Deng, Zhuokan Gu, Nan Yang, Ling Li, Xiaofeng Yue, Yawei Que, Guochang Sun, Zhengyi Wang, Jiaoyu Wang

**Affiliations:** 1 State Key Laboratory for Rice Biology, Institute of Biotechnology, Zhejiang University, Hangzhou, 310029, China; 2 State Key Laboratory Breeding Base for Zhejiang Sustainable Pest and Disease Control, Institute of Plant Protection and Microbiology, Zhejiang Academy of Agricultural Sciences, Hangzhou, 310021, China; 3 School of Agricultural and Food Sciences, Zhejiang Agriculture and Forest University, Hangzhou, 311300, China

## Abstract

Peroxisomes are required for pathogenicity in many phytopathogenic fungi, but the relationships between fungal pathogenicity and peroxisomal function are not fully understood. Here, we report the identification of a T-DNA insertional mutant C445 of *Magnaporthe oryzae*, which is defective in pathogenicity. Analysis of the mutation confirmed an insertion into the gene *MoPEX1*, which encodes a putative homologue to peroxin 1. Targeted gene deletion mutants of *MoPEX1* were nonpathogenic and were impaired in vegetative growth, conidiation, and appressorium formation. *ΔMopex1* mutants formed abnormal, less pigmented, and nonfunctional appressoria, but they were unable to penetrate plant cuticle. The *ΔMopex1* mutants were defective in the utilization of fatty acids (e.g., olive oil and Tween-20). Moreover, deletion of *MoPEX1* significantly impaired the mobilization and degradation of lipid droplets during appressorium development. Interestingly, deletion of *MoPEX1* blocked the import of peroxisomal matrix proteins. Analysis of an *M. oryzae* strain expressing GFP-MoPEX1 and RFP-PTS1 fusions revealed that MoPex1 localizes to peroxisomes. Yeast two hybrid experiments showed that MoPex1 physically interacts with MoPex6, a peroxisomal matrix protein important for fungal morphogenesis and pathogenicity. Taken together, we conclude that *MoPEX1* plays important roles in peroxisomal function and is required for infection-related morphogenesis and pathogenicity in *M. oryzae*.

Rice blast caused by the ascomycete *Magnaporthe oryzae* is one of the most serious rice diseases in most rice-growing areas worldwide. The rice blast disease is initiated when a three-cell conidium lands on the surface of a rice leaf. The conidium attaches and germinates and, subsequently forms a specialized infection structure called an appressorium at the tip of a germ tube. The appressorium attaches strongly to the rice leaf and generates enormous turgor (up to 8 MPa) by accumulating osmolytes such as glycerol, which is used to allow a narrow penetration peg to mechanically rupture the plant cuticle^1^,^2^,^3^. Eventually, the fungus spreads through the rice leaf cells and produces a visible necrotic lesion on the rice leaf surface after 3–5 days^3^,^4^.

Peroxisomes are organelles found in nearly all eukaryotic cells. They are single-membrane-bound, dynamic organelles that can change their number, size and protein composition in response to environmental conditions. In eukaryotic cells, various important metabolic processes occurr in peroxisomes. For instance, in yeasts, peroxisomes are required for metabolizing nonfermentable carbon sources such as methanol and oleate^5^,^6^,^7^,^8^,^9^. In plants, peroxisomes mediate a wide range of processes, including the glyoxylate cycle, detoxification, and the synthesis of phytohormones^10^. In humans, peroxisomes play an important role in numerous metabolic pathways, including the synthesis of plasmalogens, cholesterol, and bile acids^11^,^12^,^13^,^14^,^15^.

Peroxisomes contain two types of protein molecules, namely, membrane proteins and matrix proteins, but do not contain DNA or an independent protein synthesis machinery, so the two types of protein are encoded by nuclear genes and synthesized on free polysomes in the cytosol, which are targeted post-translationally to the peroxisomes^16^. Many integral membrane proteins are targeted to peroxisomes directly from the cytosol by the peroxisomal membrane targeting signal 1 (mPTS1), and some might be transported to peroxisomes indirectly through the ER, by mPTS2^17^,^18^,^19^. However, matrix proteins are translocated to peroxisomes by the peroxisomal targeting signals (PTSs), at least including two types: PTS1 and PTS2. PTS1 is a conserved tripeptide (S/A/C) (H/R/K) (I/L/M) at the C-terminus, while PTS2 is a nonapeptide with the consensus motif (R/K) (L/V/I) X_5_ (H/Q) (L/A), which is located near the N-terminus or at internal locations in proteins^8^,^20^. These signals on matrix proteins are recognized in the cytosol by soluble PTS receptors, Pex5 for PTS1 proteins and Pex7 for PTS2 proteins^16^,^20^,^21^,^22^.

Proteins involved in peroxisomal biogenesis are named peroxins, which are encoded by *PEX* genes^23^. Up to date, 32 different *PEX* genes have been identified^9^. Results from several experiments have indicated that fungal peroxisomes are involved in plant infection. Kimura *et al.* first reported that *Colletotrichum lagenarium PEX6* gene was required for peroxisomal function and virulence^24^. Similarly, in *M. oryzae*, Pex6-mediated peroxisome biogenesis is essential for appressorium-mediated plant infection^25^,^26^. Deletion mutants of the *MoPEX6* gene produced significantly less conidia, formed nonfunctional appressoria, and were nonpathogenic to host plants^26^. Moreover, several genes associated with peroxisome metabolism, such as *PTH2, MFP1,* and *MLS1,* have also been identified and analyzed^25^,^26^,^27^,^28^. Recently, in *M. oryzae*, it has been demonstrated that *MoPEX5* and *MoPEX7* are both required for host infection, although *MoPEX5* plays a predominant role^29^,^30^. In addition, deletion of *MoPEX19* led to metabolic deficiency involved in peroxisomes, and consequently, failure to penetrate the host^31^.

Peroxisomes are important organelles in which β-oxidation occurrs^32^. Both mitochondrial and peroxisomal β-oxidation are present in mammals. In yeasts, however, β-oxidation appears to be solely a peroxisomal process^33^. It has been found that both mitochondrial and peroxisomal β-oxidation processes occur in *Aspergillus nidulans*^34^. In *M. oryzae*, lipid body is an important storage substance in conidia. Previous experiments have demonstrated that lipid droplets are mobilized from germinating conidia and taken up by vacuoles before degrading at the onset of turgor generation^35^,^36^.

Recently, we carried out a large-scale *At*MT (*Agrobacterium tumefaciens*-mediated transformation) in *M. oryzae* to identify insertional mutants and define novel genes required for pathogenicity. Several important genes required for regulating infection and related morphogenesis have been identified via the *At*MT library. Here, we report the identification and characterization of *MoPEX1*, which putatively encodes peroxin 1 in *M. oryzae*. Our data shows that *MoPEX1* is essential for infection-related morphogenesis and pathogenicity in the rice blast fungus.

## Results

### Identification of a non-pathogenic mutant C445

**Figure 1:**
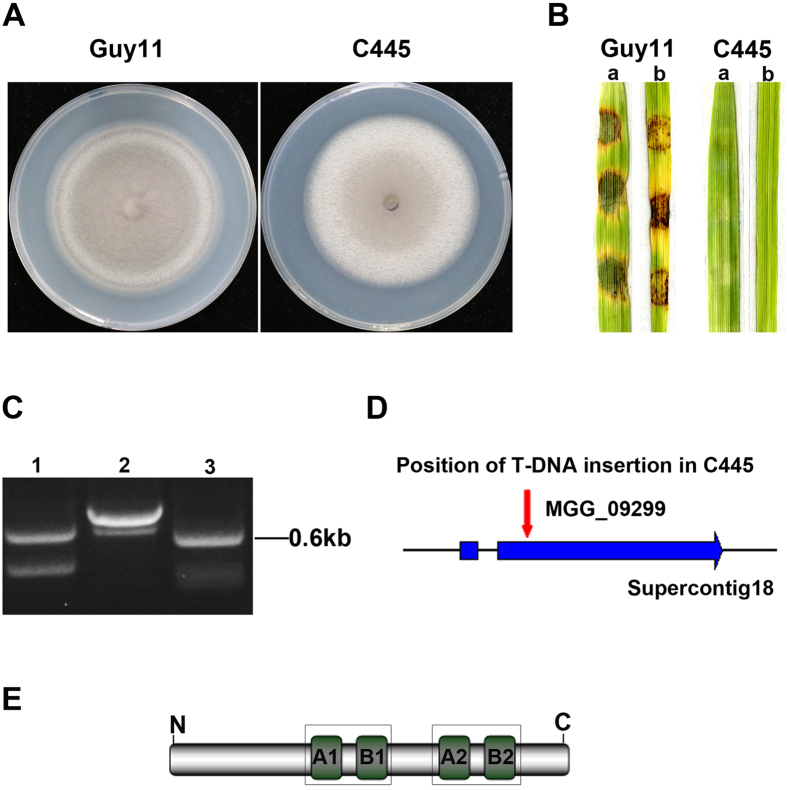
Identification of the T-DNA targeted gene *MoPEX1*. (**A**) Colonies of the wild-type strain (Guy11) and the T-DNA insertional mutant (C445). Photographs were taken after incubating on complete medium (CM) at 25 °C for 10 days. (**B**) Barley (a) and rice (b) leaf segments were inoculated with mycelial plugs from Guy11 and C445, respectively. Photographs were taken at 5 days after inoculation. (**C**) Three round PCR amplifications of the genomic DNA flanking right site of the integration T-DNA in C445. (**D**) Position of the T-DNA insertion in C445 and the structure of the locus of the predicted gene MGG_09299. A red arrow indicates the T-DNA insertion position. The thick blue arrow represents the predicted exons. (**E**) MoPex1 contains two AAA-domains. Each domain contains a Walker A and a Walker B motifs, which bind and hydrolyze ATP, respectively.

Recently, we performed *At*MT in the *M. oryzae* wild-type strain Guy11 and obtained 2347 transformants. All the transformants were first screened by inoculation of 7-day-old barley leaves using a cut-leaf assay, and a non-pathogenic mutant C445 was identified (Fig. 1A,B). Consistently, the T-DNA insertional mutant C445 was unable to cause rice blast disease (Fig. 1B). To identify the T-DNA integration sites in the mutant C445, a 0.6-kb genomic DNA flanking the left side of the integration site was obtained by thermal asymmetric interlaced polymerase chain reaction (TAIL-PCR) amplification and sequenced (Fig. 1C). Bioinformatics analysis revealed that the T-DNA in C445 was integrated at position 4280958+ on supercontig 18 of chromosome I, 536 bp downstream of the predicted translational start site, in the second exon of MGG_09299 (Fig. 1D). MGG_09299 putatively encodes a protein homologous to peroxin 1 (Pex1), a protein functioning in peroxisome biosynthesis, and was thus termed as *MoPEX1* in this work. MoPex1 shared 60%, 51%, and 38% amino acid identity with Pex1 homologs *Fusarium graminearum* FGSG_07104, *Aspergillus nidulans* ANIA_05991, and *Saccharomyces cerevisiae* YKL197C, respectively. MoPex1 is a member of the AAA-Type ATPase family. The AAA-domain comprises 200–250 amino acids and contains the Walker A and Walker B motifs, which bind and hydrolyze ATP, respectively (Fig. 1E).

### *MoPEX1* is essential for pathogenicity

To verify whether the phenotypes of C445 were truly caused by the insertion mutation, targeted deletion of *MoPEX1* was carried out using the gene replacement vector pKO9299 (see Supplementary Fig. S1A). Four independent *ΔMopex1* mutants (*ΔMopex1-*21, *ΔMopex1-*32, *ΔMopex1-*49, and *ΔMopex1-*57) were obtained from several transformation experiments. Southern blotting analysis confirmed that the correct gene replacement events had taken place in these mutants (see Supplementary Fig. S1B). The inactivation of transcription of *MoPEX1* in the selected *ΔMopex1* mutants (*ΔMopex1-*21 and *ΔMopex1-*57) was also indicated by reverse transcription-PCR (RT-PCR) analysis (see Supplementary Fig. S1C). To further verify whether the phenotypes of the null mutants were caused by gene deletion, we conducted complementary analysis. The complemented transformant *ΔMopex1*-57C was obtained by transforming the genomic DNA, covering the full length *MoPEX1* open reading frame (ORF) and a 2.0-kb promoter region, into the *ΔMopex1* mutant (*ΔMopex1-*57).

**Figure 2:**
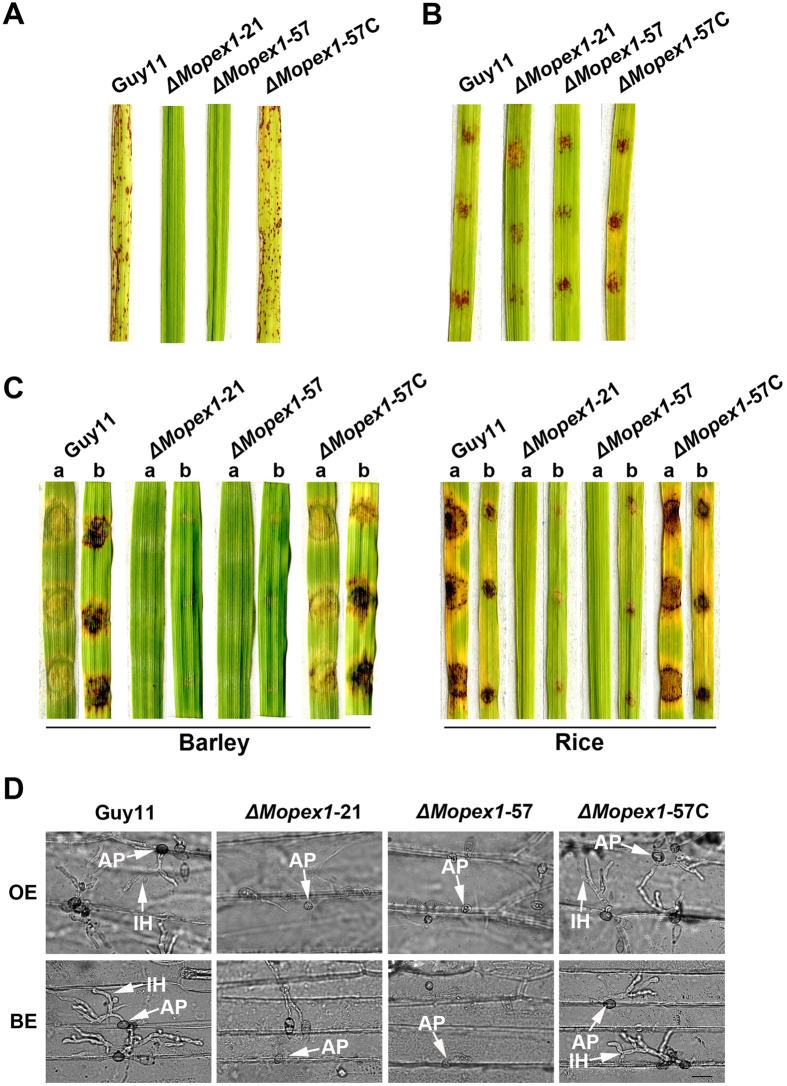
M*oPEX1* is essential for pathogenicity. (**A**) Spray-inoculation assays. Seedlings of rice were sprayed with 8 ml conidia suspension (5 × 10^4^ conidia ml^−1^) of the wild-type strain (Guy11), two *ΔMopex1* null mutants (*ΔMopex1-*21 and *ΔMopex1*-57) and the complemented strain (*ΔMopex1*-57C). (**B**) Conidia of each strain were inoculated onto rice leaf segments in the presence of 2.5% glucose solution at a concentration 1 × 10^5^ conidia ml^−1^, 10 μl per drop. (**C**) Barley and rice segments were inoculated with the mycelia of the strains. a = unwounded leaf, b = abraded leaf. (**D**) Appressorium formation and infection hyphal development on onion and barley epidermal cells at 24 and 30 hours post inoculation (hpi). AP, appressorium; IH, infectious hypha. Bars = 20 μm. Disease lesions in (**A**,**B**,**C**) were examined and photographed at 5 days post inoculation.

To investigate the role of *MoPEX1* in virulence, we carried out cut-leaf inoculation and spray-inoculation assays. Pathogenicity assays showed that both the wild-type strain and the complemented transformant caused typical rice blast lesions. Consistent with mutant C445, the *ΔMopex1* mutants were nonpathogenic on rice leaves (Fig. 2A).

Previously, it has been reported that peroxisomal fatty acid β-oxidation is vital for facilitating appressorium-mediated infection in *M. oryzae*^25^,^26^,^27^. One potential role for generated acetyl-CoA might be as a substrate for the glyoxylate cycle which feeds through reactions of the gluconeogenesis pathway to support glucan and chitin biosynthesis required for cell wall biosynthesis. Therefore, we performed rice pathogenicity assays using *ΔMopex1* mutants in the presence of 2.5% glucose solution. The results showed that the ability to cause rice blast symptoms was partly restored to the *ΔMopex1* mutants in the presence of glucose (Fig. 2B). It seems likely that addition of exogenous glucose to the *ΔMopex1* mutants can partly restore the functional competence of appressoria in this fungus.

In addition, the *ΔMopex1* mutants could form very limited lesions on abraded leaves (Fig. 2C), indicating cellular invasive growth was also impaired. Appressorium formation and infection hypha development were observed by dropping conidia suspensions of various strains on onion and barley epidermal cells. The *ΔMopex1* mutants produced non-functional appressoria that failed to elaborate any penetration pegs or infection hyphae (Fig. 2D). The defects in appressorium-mediated penetration and pathogenicity were fully restored by reintroduction of *MoPEX1* into the *ΔMopex1*-57 (Fig. 2). Thus, we conclude that *MoPEX1* is essential for appressorium-mediated penetration and pathogenicity in *M. oryzae*.

### *MoPEX1* is required for conidiation

Deletion of *MoPEX1* resulted in reduction of vegetative growth and conidiation. The *ΔMopex1* mutants (*ΔMopex1*-21 and *ΔMopex1*-57) were significantly reduced in radial growth, forming colonies with diameters of 5.97 ± 0.06 cm and 5.83 ± 0.06 cm after 10-days incubation on complement medium (CM) at 25 °C, compared with 6.87 ± 0.08 cm diameter of the wild-type strain (see Supplementary Fig. S2A,B). By examining with an optical microscope, we observed that conidiophore differentiation and conidia production were severely impaired in the *ΔMopex1* mutants (see Supplementary Fig. S2C). The ability to form asexual spores was further evaluated by carefully washing the surface of 10-day-old cultures of different strains. The *ΔMopex1*-21 and *ΔMopex1*-57 mutants produced 55.0 ± 8.66 × 10^4^ and 70.0 ± 8.66 × 10^4^ spores per plate, respectively, whereas the wild-type strain Guy11 produced 47.3 ± 0.65 × 10^6^ spores per plate (see Supplementary Fig. S2D). The defects in colonial growth and conidiation were fully restored by reintroduction of the full-length ORF of *MoPEX1* gene into the *ΔMopex1* mutants (*ΔMopex1*-57C).

Moreover, we tested the sexual reproduction of the *ΔMopex1* mutants on oatmeal agar (OMA) medium. After four weeks, numerous perithecia were observed at the junctions of the crosses, in both Guy11 × TH3 and *ΔMopex1*-57 × TH3 (see Supplementary Fig. S2E). These results indicated that *MoPEX1* is not required for sexual reproduction by *M. oryzae*.

### *MoPEX1* is required for appressorium development

**Figure 3:**
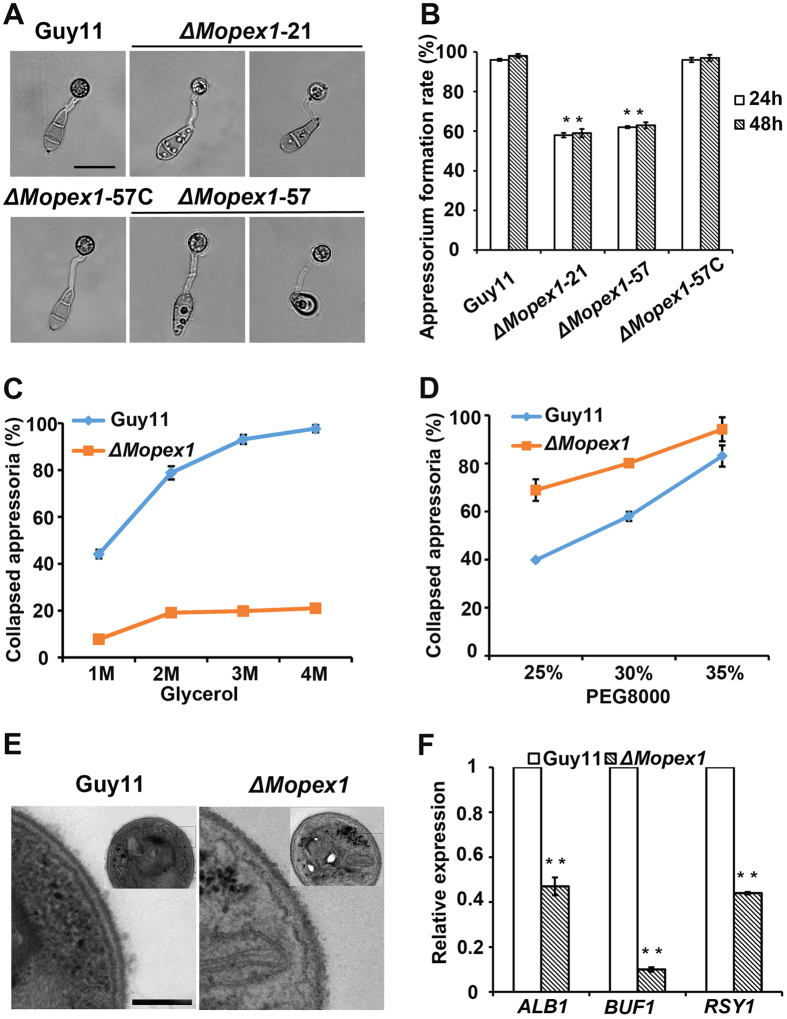
*MoPEX1* is required for appressorium development by *M. oryzae*. (**A**) The *ΔMopex1* mutants (*ΔMopex1-*21 and *ΔMopex1-*57) formed less melanized appressoria on hydrophobic GelBond film surfaces at 24 hpi compared to the wild-type strain. Bars = 20 μm. (**B**) Appressorium formation rates at 24 and 48 hpi. (**C**,**D**) Cytorrhysis tests. Gradient concentrations of glycerol: 1, 2, 3, and 4 M (**C**); PEG8000: 25%, 30% and 35% (**D)**. (**E**) Ultrastructural analysis of appressorium cell wall. Bars = 200 nm. (**F**) Expression levels of melanin biosynthesis-related genes. Means and standard deviations were calculated based on three independent experiments. Statistical difference is indicated by asterisks (*P *< 0.01).

To determine whether deletion of *MoPEX1* had an effect on appressorium formation, conidia from the *ΔMopex1* mutants were allowed to germinate on a hydrophobic surface. After incubation for 24 h, we observed that appressoria of the *ΔMopex1* mutants were abnormal under light microscopy compared to those of the wild-type strain (Fig. 3A). The appressorium formation rates of *ΔMopex1*-21 and *ΔMopex1*-57 were 58 ± 1% and 62 ± 1%, respectively, significantly lower than the rate of 96 ± 1% of the wild-type strain Guy11 (Fig. 3B). Even by 48 h, appressorium formation rates of the mutants were not significantly increased (Fig. 3B).

Appressorium-mediated penetration depends on the enormous turgor generated in appressoria, which is used to breach the rice leaf cuticle. To determine whether deletion of *MoPEX1* leads to alteration of appressorium turgor, we carried out cytorrhysis assays^1^. Conidia were allowed to form appressoria on the hydrophobic surface and then treated with different concentrations of glycerol. Surprisingly, a significant decrease in appressorial collapse was observed in the *ΔMopex1* mutants compared to the wild-type strain Guy11 (Fig. 3C). We speculated that this phenomenon may be caused by the increased permeability of glycerol molecules in the mutants. Therefore, we performed a cytorrhysis assay using polyethylene glycol 8000 (PEG8000), which has larger molecular size than glycerol, in varying concentrations. As expected, more collapsed appressoria were observed in the *ΔMopex1* mutants treated with PEG8000 compared to the wild-type strain Guy11 (Fig. 3D). These results suggested that glycerol, but not PEG8000, could cross the cell wall pores of the mutants. To determine whether MoPex1 is associated with regulation of cell wall integrity in *M. oryzae*, we exposed the *ΔMopex1* mutants to agents associated with cell wall stress, including Congo Red (CR) and sodium dodecyl sulfate (SDS). Compared to the wild-type Guy11, the mutants exhibited higher sensitivity to SDS and Congo red (see Supplementary Fig. S3), indicating that *MoPEX1* plays an important role in the cell wall integrity. To further determine the alteration of melanization of cell walls, the factor related directly to the cell wall permeability, appressoria from the *ΔMopex1* mutants were observed using transmission electron microscopy (TEM). We found that the melanin layer of the *ΔMopex1* mutants was thinner than that of the wild-type strain Guy11 (Fig. 3E). Consistently, quantitative real time PCR (qRT-PCR) analysis revealed that the expression levels of the genes *ALB1*, *BUF1* and *RSY1* involved in melanin synthesis were significantly reduced (Fig. 3F). These results suggested that *MoPEX1* was involved in appressorial melanization and turgor generation in *M. oryzae*.

Taken together, we conclude that *MoPEX1* is required for appressorium formation, morphology, and the cell wall integrity in *M. oryzae*.

### Deletion of *MoPEX1* blocks the import of peroxisome matrix proteins

**Figure 4:**
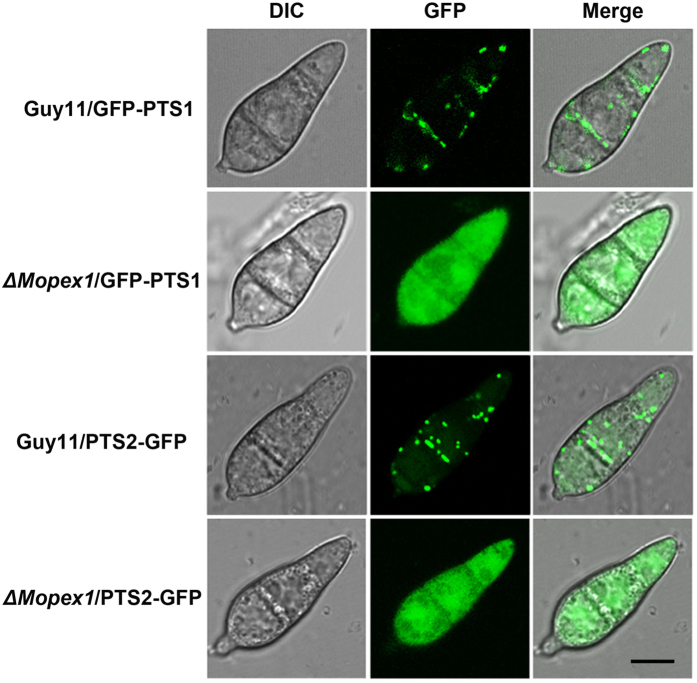
Deletion of *MoPEX1* blocked the PTS1 and PTS2 peroxisomal import pathways. The wild-type strain Guy11 and the *ΔMopex1*-57 mutant were transformed with GFP-PTS1 and PTS2-GFP, respectively. Conidia from the transformants Guy11/GFP-PTS1, *ΔMopex1*/GFP-PTS1, Guy11/PTS2-GFP, *ΔMopex1*/PTS2-GFP were observed under a confocal fluorescence microscopy. Bars = 5 μm.

Import of peroxisomal matrix proteins is dependent on the peroxisomal localization signal sequences: PTS1 and PTS2. To understand the roles of *MoPEX1* in the import process of peroxisomal matrix proteins, green fluorescence protein (GFP) fused with a C-terminal consensus motif SKL (GFP-PTS1) and GFP containing an N-terminal PTS2 (PTS2-GFP)^37^ were introduced into the wild-type strain and the *ΔMopex1-*57 mutant, respectively. Four transformants (Guy11/GFP-PTS1, *ΔMopex1*/GFP-PTS1, Guy11/PTS2-GFP, and *ΔMopex1*/PTS2-GFP) consistent with the phenotypes of their parents, in which GFP-PTS1 or PTS2-GFP was expressed, were selected. Localization of GFP fluorescence was investigated in the conidia of each strain under a confocal microscope. When GFP-PTS1 or PTS2-GFP was expressed in the wild type, punctuate fluorescence was observed in conidia (Fig. 4), indicating that the GFP-PTS1 or PTS2-GFP is recognized correctly and transported into the peroxisomes. In contrast, the GFP fluorescence of the transformants derived from the *ΔMopex1* mutant was dispersed in the cytoplasm (Fig. 4), indicating that the *ΔMopex1* mutant is defective in the import of peroxisome matrix proteins. These observations suggest that deletion of *MoPEX1* blocks the import of peroxisome matrix proteins in *M. oryzae*.

### MoPex1 localizes to peroxisomes

**Figure 5:**
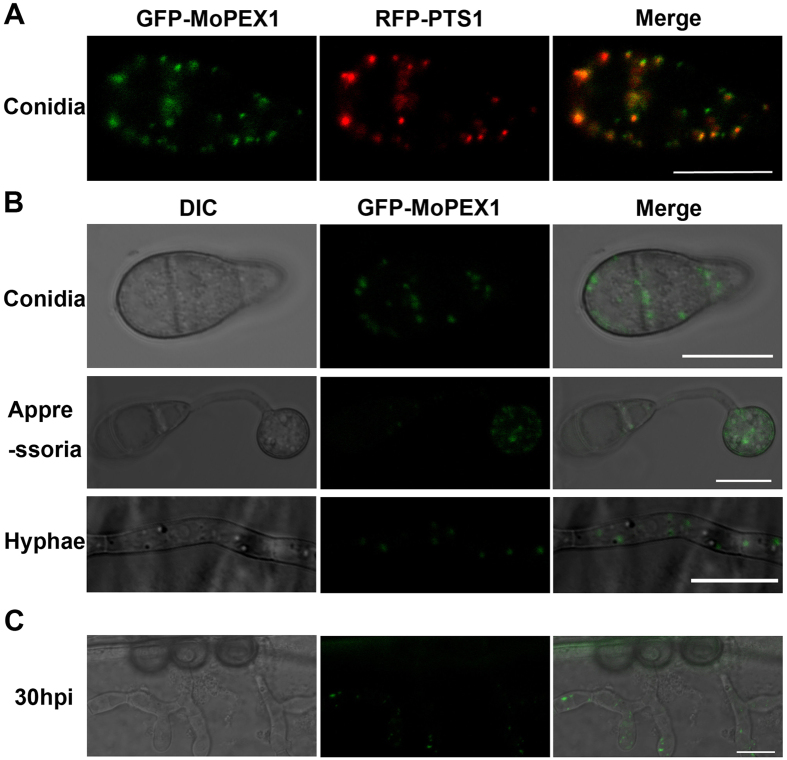
MoPex1 localizes to peroxisomes. (**A**) Fluorescence of conidia from the co-transformant strain GDW2 expressing GFP-MoPEX1 and RFP-PTS1 was observed under a confocal microscopy. Bars = 10 μm. (**B**) MoPex1 expressed in the conidia, appressoria (incubated for 12 h) and vegetative hyphae. Bars = 10 μm. (**C**) The GFP-Mopex1 expression was observed in invasive hyphae at 30 hpi. Bars = 10 μm.

In order to investigate the intracellular location of MoPex1, we performed expression analysis of the GFP-MoPex1 fusion protein. The *eGFP:MoPEX1* plasmid, in which a 2.0-kb native promoter fragment and the entire *MoPEX1* sequence were fused with *eGFP*, was constructed and then introduced into the *ΔMopex1-*57 mutant. The transformant NG9-3 showed similar phenotypes as the wild-type strain Guy11, suggesting that the *GFP-MoPEX1* fusion gene was correctly expressed. After introducing the red fluorescence protein (RFP) construct *RFP-PTS1* into the NG9-3 strain, the transformant GDW2 expressing both RFP-PTS1 and GFP-MoPEX1 was obtained. In the conidia of the GDW2 strain, GFP and RFP fluorescence showed a punctuate distribution largely at the same localization (Fig. 5A), indicating that MoPex1 localizes to peroxisomes. The temporal and spatial expression of MoPex1 was also investigated. We observed that a punctuate distribution during infection-related development including conidiation, appressorium formation, mycelial growth, and invasive growth at 30 hpi (Fig. 5B,C).

### Mobilization and degradation of lipid droplets is delayed in *ΔMopex1* mutants

**Figure 6:**
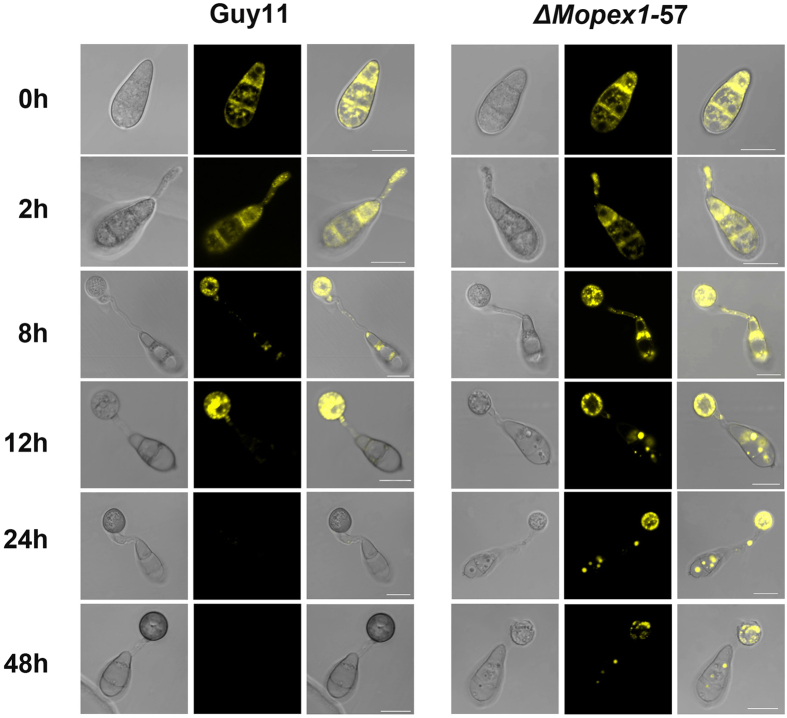
Deletion of *MoPEX1* delayed lipid mobilization and degradation during appressorium development in *M. oryzae*. Conidia of the wild-type strain Guy11 and the *ΔMopex1*-57 mutant were incubated on the hydrophobic GelBond film surfaces and allowed to form appressoria. Samples at different time points (0, 2, 8, 12, 24 and 48 h) were stained with Nile Red and observed under a confocal microscopy. Bars = 10 μm.

To identify the role of *MoPEX1* in lipid metabolism, Nile Red staining of intracellular lipid stores was carried out during appressorium morphogenesis of the *ΔMopex1* mutant and the wild-type strain (Fig. 6). In the wild type, lipid droplets were scarcely observed in the conidium at 12 h and had been completely degraded at 24 h. However, larger lipid droplets were present in the conidium of the *ΔMopex1* mutant at 24 h, indicating that mobilization of lipid droplets from conidia to appressoria was delayed in the *ΔMopex1* mutant. Even at 48 h, lipid droplets were still present in the appressoria of the *ΔMopex1* mutant and many lipid droplets still remained in the conidia (Fig. 6). These data suggested that translocation and degradation of lipid droplets were impaired by the deletion of *MoPEX1*.

### *MoPEX1* is required for fatty acid metabolism

**Figure 7:**
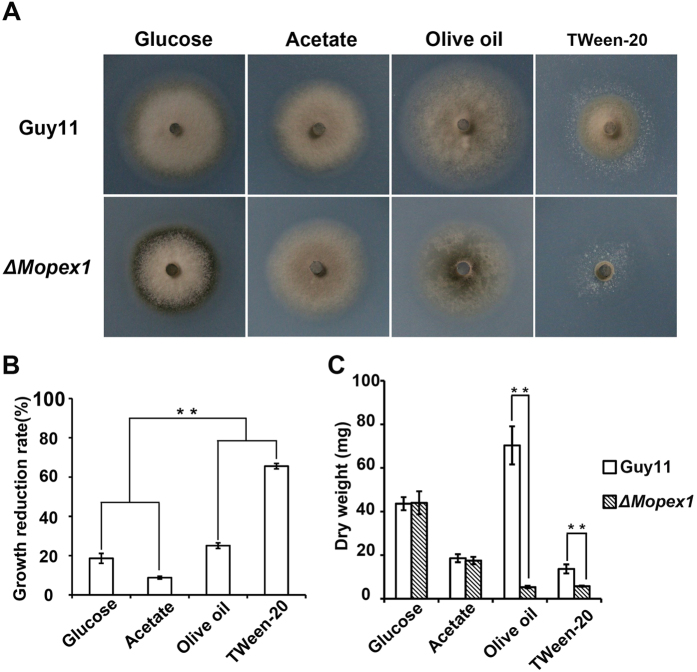
Deletion of *MoPEX1* affects lipid utilization. Lipid utilization by the wild-type strain Guy11 and the *ΔMopex1*-57 mutant. The strains were cultured on minimal media with glucose (1%), sodium acetate (50 mM), olive oil (0.2%) or Tween-20 (0.5%) as the sole carbon source for 10 days. (**B**) Growth reduction rates of colony growth on different media. (**C**) The wild-type strain Guy11 and the *ΔMopex1*-57 mutant were inoculated in liquid minimal media with different carbon sources as the sole carbon source at 28 °C for 5 days. Means and standard deviations were calculated based on three independent experiments. Statistical difference is indicated by asterisks (*P* < 0.01).

In fungi, the peroxisomes are important organelles in which fatty acid β-oxidation occurs. In the Nile Red staining experiments, we observed more lipid droplets in the conidia and appressoria of the *ΔMopex1-*57 mutant than in those of the wild type. In order to clarify the effect of the deletion of *MoPEX1* on fatty acid metabolism, vegetative growth on minimal medium (MM) with glucose (1%), sodium acetate (50 mM), olive oil (0.2%), or Tween-20 (0.5%) as the sole carbon source was assessed. After incubation for 10 days, the growth reduction rate of the *ΔMopex1*-57 mutant grown with sodium acetate was 8.8%, lower than the rate of 18.6% of the *ΔMopex1*-57 mutant grown with glucose. However, the growth of the *ΔMopex1-*57 mutant was significantly inhibited on the medium supplemented with olive oil or Tween-20 (Fig. 7A,B). The reduction rates of the *ΔMopex1-*57 mutant under olive oil or Tween-20 reached 25.1% and 65.6%, respectively. Similar results were obtained for the *ΔMopex1-*21 mutants under these conditions.

We further examined the dry weight of the mycelia obtained from Guy11 and the *ΔMopex1* mutants by incubating them in liquid MM with glucose, sodium acetate, olive oil, or Tween-20 as the sole carbon source. After 5 days at 28 °C, there was no obvious difference in weight when grown on glucose and acetate. In contrast, the dry weight of the *ΔMopex1* mutants was significantly reduced when grown under olive oil or Tween-20 as the sole carbon source (Fig. 7C), compared to Guy11, indicating that the *ΔMopex1* mutants are defective in the utilization of fatty acids. Taken together, these results suggested that *MoPEX1* is required for fatty acid metabolism in *M. oryzae*.

### MoPex1 physically interacts with MoPex6

**Figure 8:**
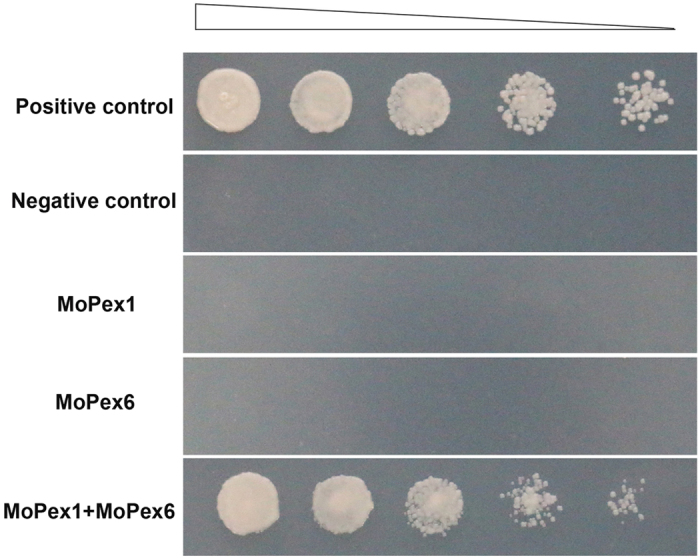
MoPex1 interacts with MoPex6. The vectors pGBKT7/pGADT7 (negative control), pGBKT7-53/pGADT7-T (positive control), pGAD-Pex1/pGBKT7, pGBK-Pex6/pGADT7, pGAD-Pex1/pGBK-Pex6 were co-transformed to the yeast strain AH109, respectively. The Leu+ and Trp+ yeast transformants were assayed for growth on SD/–Trp/–Leu/–His/–Ade medium at specified concentrations 1 × 10^5^, 1 × 10^4^, 1 × 10^3^, and 1 × 10^2^ cells per 10 μl droplet.

In *Pichia pastoris*, it has been first reported that Pex1 interacts with Pex6^38^ and the interaction of Pex1 and Pex6 was confirmed in *Homo sapiens*^39^, *Hansenula polymorpha*^40^, and *Saccharomyces cerevisiae*^41^. To determine whether MoPex1 interacts with MoPex6 in *M. oryzae*, we carried out yeast two hybrid (Y2H) experiments. The cDNA of *MoPEX1* was cloned into pGADT7 as the prey vector pGADT7-PEX1. The cDNA of *MoPEX6* was cloned into pGBKT7 as the bait vector pGBKT7-PEX6. The resulting prey and bait vectors were co-transformed into the yeast strain AH109. The Leu+ and Trp+ yeast transformants were isolated and assayed for growth on SD/–Trp/–Leu/–His/–Ade medium. The results provided evidence that MoPex1 physically interacts with MoPex6 (Fig. 8).

## Discussion

*M. oryzae* can mechanically breach the rice leaf surface using enormous turgor generated in appressoria by accumulating osmolytes such as glycerol^1^,^2^,^3^. The degradation of lipids during appressorium formation is an efficient means of rapidly producing glycerol and also leads to production of fatty acids for β-oxidation^35^, which is the principal means by which fatty acids are degraded. Previous studies have provided the evidence that either peroxisome biosynthesis or fatty acid β-oxidation is a prerequisite for appressorium-mediated plant infection in *M. oryzae*^25^,^26^. In this study, we identified and characterized *MoPEX1*, homologous to *PEX1* in yeast, which is important for peroxisomal matrix protein import and the degradation of the lipid body. Although Pex1 orthologs are well conserved in filamentous ascomycetes, but, to our knowledge, none of them has been functionally characterized previously. In this study, our data showed that *ΔMopex1* mutants were defective in infection-related morphogenesis and were nonpathogenic. We found that appressoria produced by the mutants were non-functional and unable to penetrate plant cuticle. Moreover, under light microscopy and TEM, appressoria of the *ΔMopex1* mutants were found to be abnormal and less pigmented compared to those of the wild-type strain. Therefore, *MoPEX1* is essential for appressorium-mediated penetration and pathogenicity in *M. oryzae*.

Deletion of *MoPEX1* may impair peroxisome synthesis and fatty acid β-oxidation, as well as causing reduced acetyl CoA production. There are three possible aspects that determine plant infection behavior of the *ΔMopex1* mutants. First, acetyl CoA may not be available for glyoxylate cycle and gluconeogenesis to provide glucan and chitin biosynthesis, which are required for cell wall generation^4^. This point is supported by the evidence that addition of exogenous glucose to appressoria of *ΔMopex1* mutants partially restored their ability to cause disease (Fig. 2B). Second, deficiency of acetyl CoA may affect appressorium turgor generation in the *ΔMopex1* mutants (Fig. 3C,D), because acetyl CoA from fatty acid β-oxidation can provide precursors for glycerol production through the glyoxylate cycle and gluconeogenesis. Third, lower level of the resulting acetyl CoA from β-oxidation may affect melanin biosynthesis via the dihydroxynaphthalene pathway. Appressorium melanization is essential for plant infection by *M. oryzae*^1^,^2^,^4^,^42^. In the *ΔMopex1* mutants, appressoria showed a thinner melanin layer and the expression levels of melanin biosynthesis-related genes were significantly reduced (Fig. 3EF).

Taken together, these deficiencies caused by low levels of acetyl CoA may lead to the loss of pathogenicity in the *ΔMopex1* mutants. We also noted that the *ΔMopex1* mutants formed very limited lesions on wounded rice leaves compared to the wild-type strain (Fig. 2C). Therefore, *MoPEX1* is important not only for initial infection but also for tissue colonization *in M. oryzae*.

Peroxisome matrix proteins are translocated to peroxisomes by targeting signals (PTS1 or PTS2). Analysis by GFP-PTS1 expression indicated that the PTS1-containing matrix protein import was blocked in the *ΔClpex6* mutants in *C. Lagenarium*^24^. In *M. oryzae*, *MoPEX5* and *MoPEX6* had been identified to participate in PTS1 matrix protein import^26^,^29^. *MoPEX7* contributes to the import of PTS2 matrix proteins^30^. Recently, it has been reported that the imports of PTS1- and PTS2-containing matrix proteins were impaired in the *ΔMopex19* mutants^31^. In this study, we compared the distribution of GFP-PTS1 and PTS2-GFP in the wild-type strain and *ΔMopex1* mutants. In the cells of the *ΔMopex1* mutants expressing GFP-PTS1 and PTS2-GFP, diffuse fluorescence was observed throughout the cytoplasm (Fig. 4). This indicated that *MoPEX1*, like *MoPEX5*, *6*, *7*, and *19*, plays an important role in peroxisome matrix protein import in *M. oryzae*.

Moreover, we also transformed GFP-PMP47, a fusion representing the integral peroxisomal membrane proteins^31^, into the wild-type strain and *ΔMopex1* mutants. Although the florescence was generally in punctate pattern, diffuse florescence could be detected in the conidia of the *ΔMopex1*/GFP-PMP47, in contrast to the absolute punctate pattern in the wild type. We also noted that the punctate GFP fluorescence in the hyphae was seemingly bigger compared to the wild type/GFP-PMP47 (see Supplementary Fig. S4). These results imply that peroxisome membrane protein import was also partially affected by the deletion of *MoPEX1.*

Degradation of lipid droplets is catalyzed by triacylglycerol lipase, producing glycerol and fatty acids for β-oxidation. Peroxisomes are important organelles wherein β-oxidation occurs. In *C. lagenarium*, the deficiency of peroxisomal function in the *ΔClpex6* mutants leads to its inability to utilize long-chain fatty acids^24^,^25^. In *M. oryzae,* the peroxisome-related mutants, *ΔMopex5, ΔMopex6, and ΔMopex7,* were unable to utilize long-chain fatty acids as sole carbon sources and *ΔMopex19* mutants showed severe growth reduction when cultured with long-chain fatty acids^26^,^29^,^30^,^31^. Moreover, yeast cells deficient in Pex1 also show a characteristic phenotype with the inability to grow on oleic acid as sole carbon source^43^,^44^. Similar to these *pex* mutants, *ΔMopex1* mutants were also defective in lipid utilization, while the glucose and acetate utilization was unaffected in the *ΔMopex1* mutants. In addition, the translocation and degradation of lipid droplets were impaired by the deletion of *MoPEX1*. This indicates that *MoPEX1* plays an important role in fatty acids metabolism.

In yeasts, Pex1 interacts with Pex6 to form a complex that participates in Pex5 recycling^45^,^46^. Yeast two hybrid experiments (Fig. 8) indicated that MoPex1 physically interacts with MoPex6. Furthermore, the expression level of *MoPEX6* in *ΔMopex1* mutants were analysed by qRT-PCR. The results exhibited that the expression level of *MoPEX6* in mycelia and appressoria of the *ΔMopex1* mutant was up-regulated (see Supplementary Fig. S5). However, the exact relationship between *MoPEX1* and *MoPEX6* or other peroxisome genes will be explored in future.

## Materials and Methods

### Strains, growth conditions, and nucleic acid manipulation

All mutants described in this study were generated from *M. oryzae* wild-type strain Guy11. Standard growth and storage procedures for fungal strains were performed, as described previously^47^. *Agrobacterium tumefaciens* AGL1 was used for T-DNA insertional transformation. *Escherichia coli* strain DH-5α was used for routine bacterial transformations and maintenance of various plasmids in this study. All the strains used in this study were listed in Supplementary Table S1. Genomic DNAs were extracted from vegetative hyphae using the cetyltrimethyl ammonium bromide (CTAB) protocol^48^. Southern blot analysis was performed by the digoxigenin (DIG) high prime DNA labeling and Detection starter Kit II (Roche, Mannheim, Germany). General procedures for nucleic acid analysis followed standard protocols^49^.

### Fungal growth, sporulation, and appressorium formation

Vegetative growth was assessed by measurement of colony diameter in plate cultures of *M. oryzae* grown on CM medium at 25 °C for 10 days. Conidial development was assessed by harvesting conidia from the surface of 10-day-old plate cultures and determining the concentration of the resulting conidial suspension using a haemocytometer (Corning). Appressorium formation was measured on hydrophobic coverslips at 25 °C for 24 h and 48 h. The percentage of appressorium formation was determined by microscopic examination of at least 100 conidia or appressoria. Each test was repeated at least three times.

### Pathogenicity assay

Two-week-old seedlings of the rice cultivar ‘CO39’ and 7-day-old seedlings of the barley cultivar ‘Golden Promise’ were used for infection assays. For cut-leaf assays, the mycelial plugs from 10-day-old CM cultures were placed onto the leaf surface and placed in plastic plates containing wet filters. Wounded leaves were prepared by removing the surface cuticle by abrasion with an emery board, as described previously^26^. For spray-inoculation assays, conidial suspensions with a concentration of 5 × 10^4^ conidia ml^−1^, diluted in 0.05% Tween-20, were spray-inoculated onto plant leaves. For inoculation of onion and barley epidermis surfaces *in vitro*, conidia from 10-day-old CM cultures were carefully inoculated on onion and barley epidermis surfaces with 1 × 10^5^ conidia ml^−1^ and incubated at 25 °C in the dark.

### Construction of vectors and fungal transformation

For generating the *MoPEX1* gene replacement vector pKO9299, the 1.5-kb upstream and 1.5-kb downstream sequences of *MoPEX1* were amplified with primer pairs LB F/ LB R and RB F/ RB R, respectively. The PCR products were cloned into pKOV21 vector to generate pKO9299. Then, pKO9299 was transformed into Guy11 for generating homologous recombinants.

For construction of the complementation vector (N-terminal GFP tagging vector) pGFP-MoPEX1, a 2.0-kb native promoter region and 3.8-kb full length region of the *MoPEX1* gene were amplified with primer pairs 9299nGFP F/promGFP R and 9299cGFP F/9299GFP R, respectively. The 0.7-kb *eGFP* coding sequence was amplified with primer pairs GFP F/GFP R. Three PCR products were cloned into pCB1532 to create pNG9299, according to the manufacturer’s instructions of One Step Cloning Kit (Vazyme, Nanjing, China). The resulting plasmid was transformed into the *ΔMopex1* mutant (*ΔMopex1*-57).

### Quantitative RT-PCR analysis and TEM

Total RNA was extracted using PureLink RNA Mini Kit (Invitrogen, USA) from mycelia cultured in liquid CM for 2 days or from appressoria formed on barley epidermis for 24 h. Synthesis of the first strand complementary DNA (cDNA) and qRT-PCR were performed as described previously^50^. The relative expression level of each gene was calculated using the 2^−ΔΔCT^ method^51^. β-tubulin (MGG_00604) was used as an endogenous reference. All the primers used for qRT-PCR assays are listed in Supplementary Table S2. Means and standard deviations were calculated based on three independent experiments. Appressoria formed on sterile onion epidermis for 24 h were processed for TEM^52^.

### Mobilization of lipid droplets during appressorium development

Conidia of tested *M. oryzae* strains were harvested from the surface of 10-day-old plate cultures. Conidial suspensions with a concentration of 5 × 10^4^ conidia ml^−1^ were incubated on hydrophobic GelBond and incubated at 25 °C. Lipid droplets in the germinating conidia, germ tubes, and appressoria of Guy11 and the *ΔMopex1* mutants were visualized by staining with Nile Red solution consisting 50 mM Tris-maleate buffer (pH 7.5), 20 mg ml^−1^ polyvinylpyrrolidone and 2.5 μg ml^−1^ Nile Red Oxazone^35^,^53^. Microscopy was performed using a Zeiss Lsm 780 inverted confocal laser scanning microscope at intervals.

### Y2H assay

Y2H assay was carried out according to the instructions of BD Matchmaker Library Construction & Screening Kits (Clontech, PaloAlto, CA, U.S.A.). The full-length cDNAs of *MoPEX1* and *MoPEX6* were amplified with the primer pairs 9299 AD F/9299 AD R and PEX6 BK F/PEX6 BK R, respectively. The cDNA of *MoPEX1* was cloned into pGADT7 as the prey vector pGADT7-PEX1. The cDNA of *MoPEX6* was cloned into pGBKT7 as the bait vector pGBKT7-PEX6. The resulting pGADT7-MoPEX1 and each bait vector were co-transformed into yeast strain AH109. The resulting bait vector and empty prey vector were also co-transformed into yeast strain AH109. The Leu+ and Trp+ yeast transformants were isolated and assayed for growth on SD/–Trp/–Leu/–His/–Ade medium. Yeast strains for positive and negative controls were from the Kit.

## Additional Information

**How to cite this article**: Deng, S. *et al.* Identification and characterization of the peroxin 1 gene *MoPEX1* required for infection-related morphogenesis and pathogenicity in *Magnaporthe oryzae. Sci. Rep.* **6**, 36292; doi: 10.1038/srep36292 (2016).

**Publisher’s note:** Springer Nature remains neutral with regard to jurisdictional claims in published maps and institutional affiliations.

## Supplementary Material

Supplementary Information
