# Supplementary material for: Identification and characterization of the peroxin 1 gene MoPEX1 required for infection-related morphogenesis and pathogenicity in Magnaporthe oryzae
Source: Sci Rep. 2016 Nov 8;6:36292. doi: 10.1038/srep36292 (PMC5099783; doi:10.1038/srep36292)
Supplement: Supplementary Information [file srep36292-s1.pdf]

Identification and characterization of the peroxin 1 gene *MoPEX1* required for infection-related morphogenesis and pathogenicity in *Magnaporthe oryzae*

Shuzhen Deng[1], Zhuokan Gu[2], Nan Yang[1], Ling Li[3], Xiaofeng Yue[1], Yawei Que[1], Guochang Sun[2], Zhengyi Wang[1*] and Jiaoyu Wang[2*]

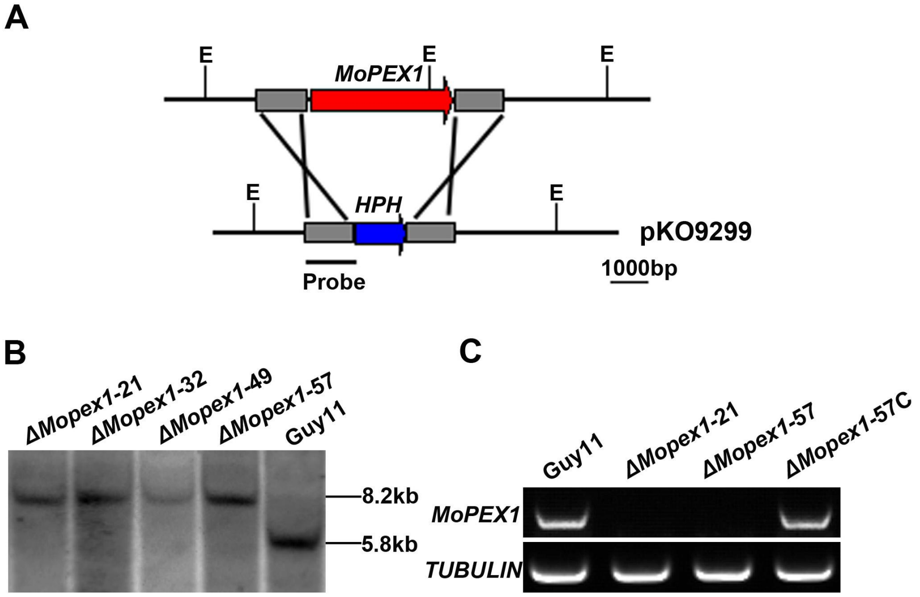

Figure S1. Targeted gene replacement of *MoPEX1*.

(**A**). Construction of the knockout vector pKO9299 and targeted gene replacement of *MoPEX1*. E : *Eco*RV. (**B**). Southern blot analysis. The genomic DNAs of the wild-type strain Guy11 and four potential *ΔMopex1* null mutants were digested with *Eco*RV and then probed with a 1.5-kb fragment amplified with the primers LB-F and LB-R. (**C**). RT-PCR was used to confirm the loss of *MoPEX1* transcripts from the two *ΔMopex1* null mutants. Expression of *TUBULIN* (MGG_00604) encoding β-tubulin was used as a control.

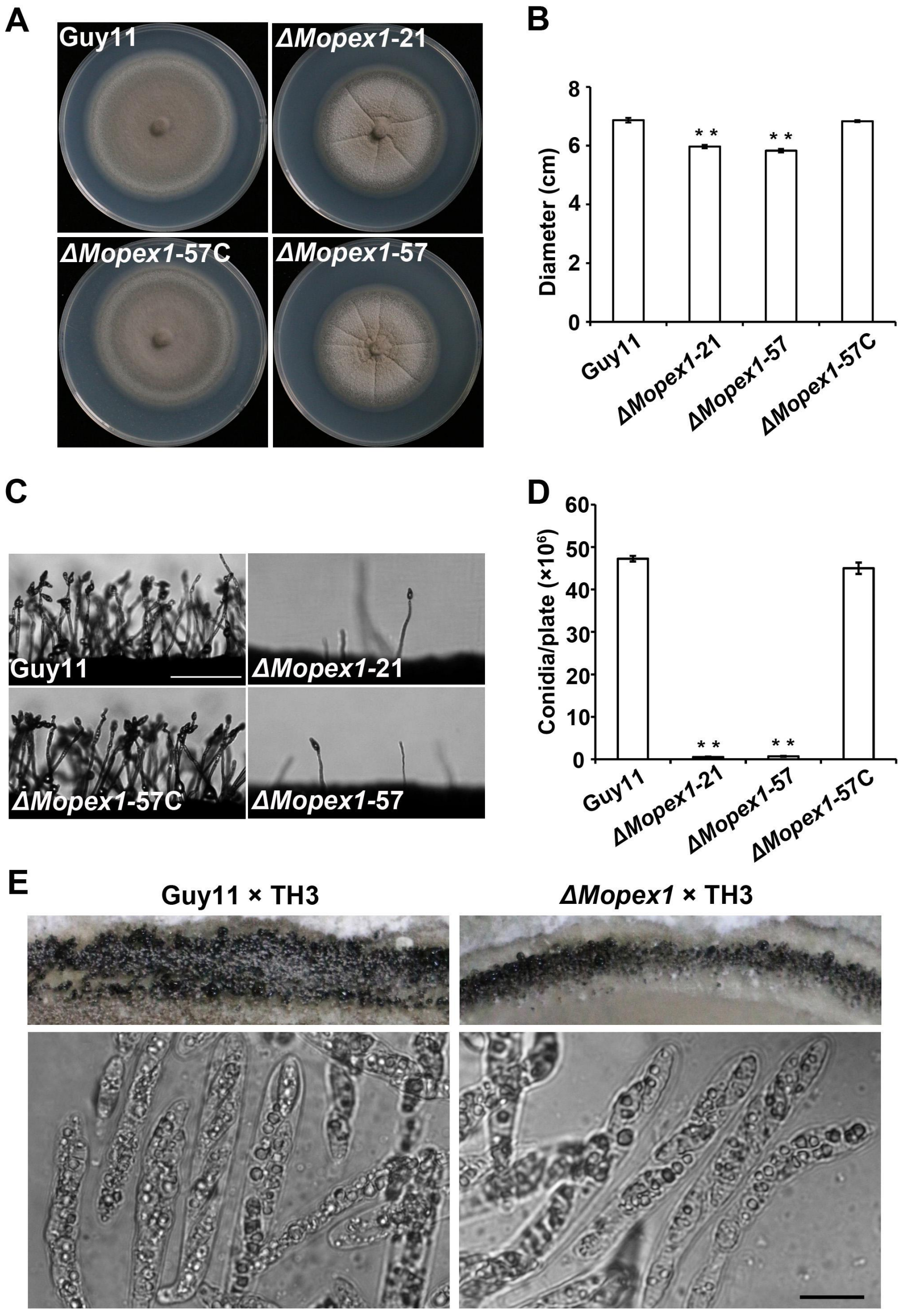
A
Guy11
ΔMopex1-21
ΔMopex1-57C
ΔMopex1-57
B
Diameter (cm)
0
2
4
6
8
Guy11
ΔMopex1-21
ΔMopex1-57
ΔMopex1-57C
C
Guy11
ΔMopex1-21
ΔMopex1-57C
ΔMopex1-57
D
Conidia/plate (×10⁶)
0
10
20
30
40
50
60
Guy11
ΔMopex1-21
ΔMopex1-57
ΔMopex1-57C
E
Guy11 × TH3
ΔMopex1 × TH3

Figure S2. ***MoPEX1* is required for conidiation.**

(**A**). Colonial morphology of the wild-type strain (Guy11), two *ΔMopex1* null mutants (*ΔMopex1*-21 and *ΔMopex1*-57) and the complemented strain (*ΔMopex1*-57C). Photographs were taken after incubating on complete medium (CM) at 25 °C for 10 days. (**B**). Bar chart showing the colony diameters of the strains. (**C**). Microscopic observation of conidial development. Aerial hyphae and conidiophores were significantly reduced in the *ΔMopex1* null mutants. Bars = 100 μm. (**D**). Bar chart showing the conidial production. Conidia were harvested from 10-day-old (CM) cultures of the strains. (**E**). Fertility assay. Both Guy11 × TH3 and *ΔMopex1*-57 × TH3 formed numerous perithecia at the junctions on OMA medium after 4-week incubation in an inductive condition. Bars = 20 μm.

Means and standard deviations were calculated based on three independent experiments.

Statistical difference is indicated by asterisks ($P < 0.01$).

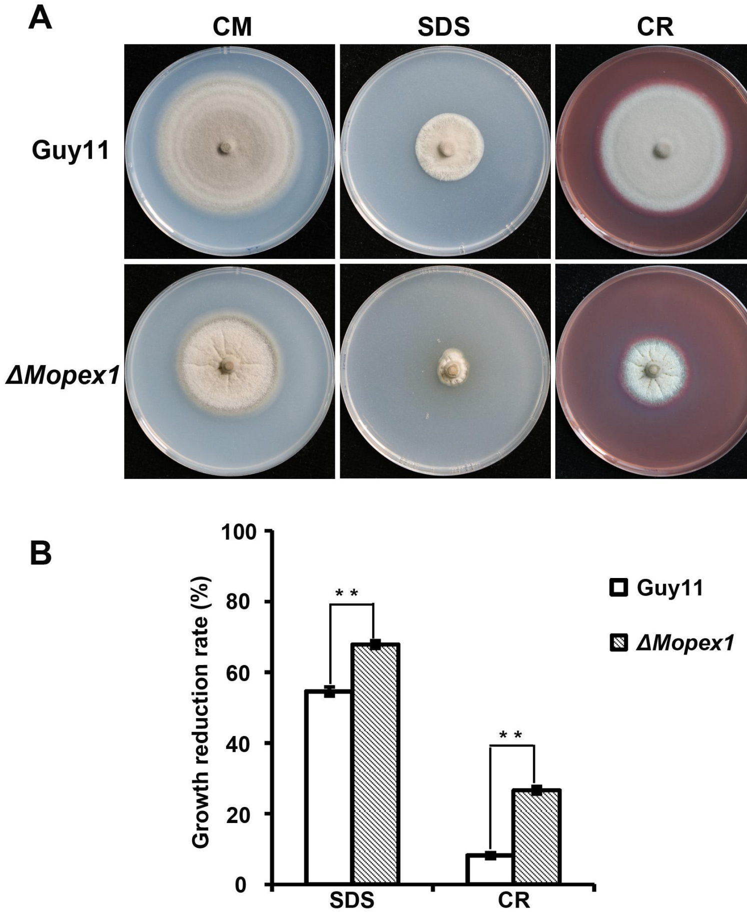

Figure S3. Cell wall integrity test of the wild-type strain Guy11 and the *ΔMopex1* mutant.

(**A**). The wild type Guy11 and the *ΔMopex1*-57 mutant were cultured on CM and CM supplied with 0.01% SDS or 200 μg ml^{-1} Congo Red (CR) for 10 days. (**B**). Growth reduction rates of colony growth on CM supplied with SDS or Congo Red (CR).

Means and standard deviations were calculated based on three independent experiments.

Statistical difference is indicated by asterisks ($P < 0.01$).

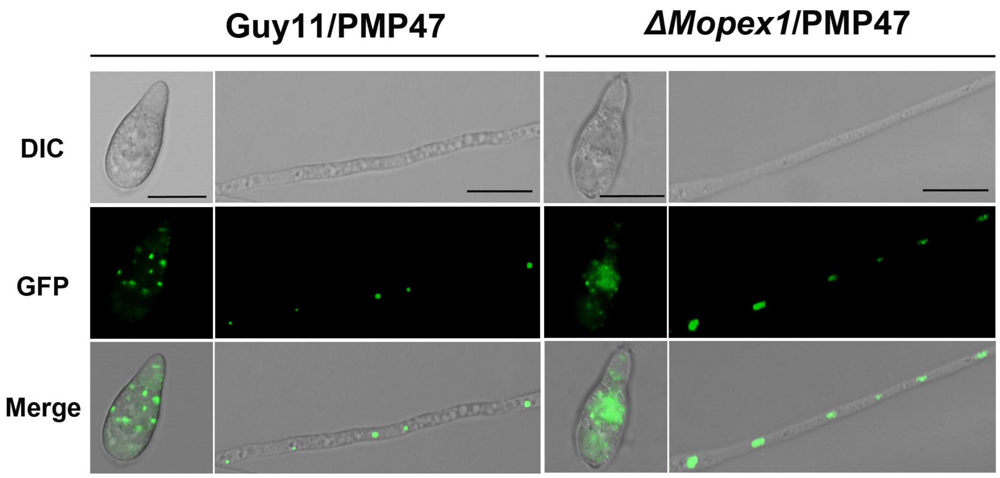

Figure S4. Distribution of the PMPs in the *ΔMopex1* mutant.

The wild-type strain Guy11 and the *ΔMopex1*-57 mutant were transformed with GFP-PMP47. Conidia and hyphae from the transformants Guy11/GFP-PMP47 and *ΔMopex1*/GFP-PMP47 were observed under a confocal fluorescence microscopy. Bars = 10 μm.

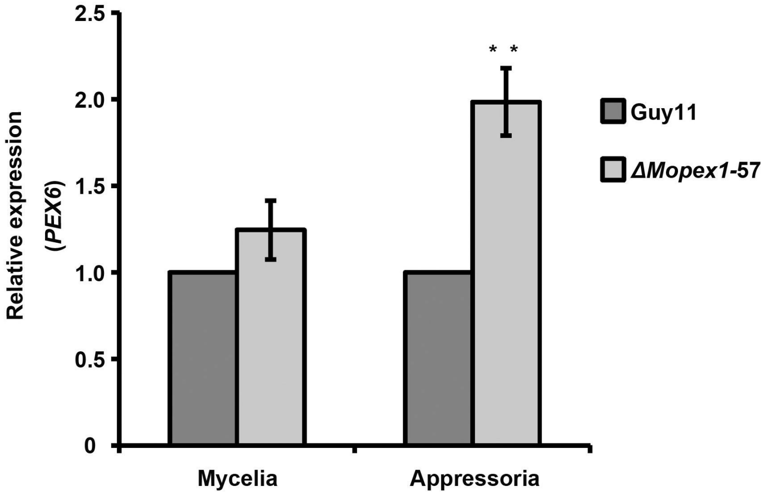

Figure S5. Expression of *MoPEX6* in the wild-type strain Guy11 and the *ΔMopex1* mutant.

Quantitative RT-PCR analysis of relative expression of *MoPEX6* in the wild-type strain Guy11 and the *ΔMopex1*-57 mutant. RNA samples were extracted from mycelia (2-day-old CM liquid culture) and appressoria formed on barley epidermis for 24 h.

Means and standard deviations were calculated based on three independent experiments. Statistical difference is indicated by asterisks ($P < 0.01$).

Table S1. Strains used in this study

Name	Brief description
Guy11	The wild-type strain of *Magnaporthe oryzae*
C445	The T-DNA insertional mutant
TH3	A standard tester strain for fertility assay
ΔMopex1-21	The *ΔMopex1* null mutant
ΔMopex1-32	The *ΔMopex1* null mutant
ΔMopex1-49	The *ΔMopex1* null mutant
ΔMopex1-57	The *ΔMopex1* null mutant
ΔMopex1-57C	The complemented strain
Guy11/GFP-PTS1	The wild-type strain expressing GFP-PTS1
ΔMopex1/GFP-PTS1	The *ΔMopex1* mutant expressing GFP-PTS1
Guy11/PTS2-GFP	The wild-type strain expressing PTS2-GFP
ΔMopex1/PTS2-GFP	The *ΔMopex1* mutant expressing PTS2-GFP
GDW2	The strain expressing GFP-MoPEX1 and RFP-PTS1
Guy11/GFP-PMP47	The wild-type strain expressing GFP-PMP47
ΔMopex1/GFP-PMP47	The *ΔMopex1* mutant expressing GFP-PMP47
AGL1	*Agrobacterium tumefaciens*
DH-5α	*Escherichia coli*

Table S2. Primers used in this study

Name	Sequence (5'-3')	Usage
ATMThb F	TCCCCCGGGCTGCAGGAATTCCCTCGTGATGATACCGCTA	Complementation
ATMThb R	GATAAGCTTGATATCGAATTCGATATACACAAACAGTTAGG	of C445
LB F	GGATCCCCCGGGCTGCAGGAATTCAGCACATCAAGAACATCGTC	*MoPEX1*
LB R	CCTTCAATATCAGTTATCGAATTCAACTAGTTCTAGCAGATCATG	knockout
RB F	CTTATCGATACCGTCGACCTCGAGTGTCTTAACCTAACTGTTTG	
RB R	GGTACCGGGCCCCCCCTCGAGAGGCGCTAGCTAGTTTGAC	
check up	GCAGAGAATGGTAAGTGTCC	Confirming the
check down	CGCCGAGTATGTCAACGTC	*ΔMopex1*
Hph up	GACAGACGTCGCGGTGAGTT	mutants
Hph down	GTCCGAGGGCAAAGAAATAG	
Tublin F1	GTTCACCTTCAGACCGG	
Tublin R1	GAGATCGACGAGGACAG	
PSZ1 SF4	ACCACACCACTTACTTTACC	
RT-SR	CGAGATCGTC CAGTATCAC	
9299nGFP F	GGATCCCCCGGGCTGCAGGAATTCTGTATAAGGAGTAGGCAA	*MoPEX1* native
promGFP R	TCCTCGCCCTTGCTCACCATTATGTGTGTGTGTGTCTTGA	promoter
GFP F	ATGGTGAGCAAGGGCGAGGA	*MoPEX1*
GFP R	CTTGTACAGCTCGTCCATGC	N-terminal GFP
9299cGFP F	GCATGGACGAGCTGTACAAGGCTCCACGAAAGAACGGGC	fusion
9299GFP R	GATAAGCTTGATATCGAATTCAGACTCCTGGTGGGCTGGC	
9299 AD F	GGCCATGGAGGCCAGTGAATTCATGGCTCCACGAAAGAACGG	*MoPEX1* cDNA
9299 AD R	TGCCCACCCGGGTGGAATTCCTACATCAAGCTAGAACGT	amplification
PEX6 BK F	GGCCATGGAGGCCGAATTCATGGACCCGGCGCAAACCC	*MoPEX6* cDNA
PEX6 BK R	TCGACGGATCCCCGGGAATCCTCAATCGTACAAGCCCTCAT	amplification
βtub-q-F	CATACGGTGACCTGAACTAC	qRT-PCR analysis
βtub-q-R	CCATGAAGAAGTGCAGACG	

ALB1-q-F	TGACACCTTCCTCAACACC
ALB1-q-R	CGAGCCAGATTTAAGCAGCC
BUF1-q-F	TACAAGCACCTCGAGATTGG
BUF1-q-R	CAGTAATCTTCTTGTCGGCC
RSY1-q-F	TTCCTCGACAAGCTCTGG
RSY1-q-R	GTGTCCTTGTACCTCTGGTG
PEX6-q-F	CAATTTGTGAACCGGCTCC
PEX6-q-R	ATATCGCGGCATGTGTCGG
